# Laparoscopic bladder diverticulectomy in a child with situs inversus totalis: A case report and literature review

**DOI:** 10.3389/fsurg.2022.1009949

**Published:** 2022-10-14

**Authors:** Jitao Chen, Fei Liu, Jie Tian, Mingfeng Xiang

**Affiliations:** Department of Urology, The Second Affiliated Hospital of Nanchang University, Nanchang, China

**Keywords:** situs inversus totalis, child, bladder diverticulum, laparoscopy, laterality defects, genetic variation

## Abstract

Situs inversus totalis (SIT) is a rare internal laterality disorder characterized by the mirror arrangement of organs. Multiple gene mutations and maternal environmental factors are thought to cause this variation. It is usually challenging to perform laparoscopic surgery in these cases. Bladder diverticulum is uncommon in children, with an incidence of 1.7%. We report a 14-year-old male patient who was admitted to our department because of lower abdominal pain and frequent urination. A series of examinations confirmed the rare combination of giant bladder diverticulum and SIT. After extensive preoperative discussion, we performed laparoscopic bladder diverticulectomy. The operation was successful. To the best of our knowledge, this is the first report of successful laparoscopic bladder surgery on a case of SIT. This article summarizes the key technical points and the difficulties of performing this kind of operation. In addition, during the process of reviewing the literature, we found that SIT often coexists with some high-risk factors for bladder diverticulum in some rare syndromes. It is helpful to further understand and provide experience in the diagnosis and treatment of the rare combination of bladder diverticulum and SIT in children.

## Introduction

Situs inversus totalis (SIT) is a rare phenomenon of abnormal physical development, and the unique mirror localization of the organs has aroused the interest of many surgeons. Bailie first described SIT in 1793. Statistics in recent years show that there is approximately one mirror person for every 6,000–8,000 people (1, 2).

Bladder diverticulum is uncommon in children, with an incidence of 1.7%. Compared with adults, where it is caused by bladder outlet obstruction, bladder diverticulum in children is often congenital and accompanied by vesicoureteral reflux. Pathological specimens often contain dysplastic muscles (3). The combination of giant bladder diverticulum and SIT is rare.

Previous studies have reported the use of laparoscopy to treat other organ lesions in patients with SIT, but this is the first report of laparoscopic bladder diverticulectomy in SIT. In addition, this paper discusses the diagnosis and treatment of this rare disease combination, especially for bladder diverticula not caused by benign prostatic hyperplasia.

## Case report

A 14-year-old child was admitted to our hospital in January 2022 with lower abdominal pain and frequent urination. According to the patient's own recollection, he had frequent urination and intermittent abdominal pain 3 months before admission, sometimes accompanied by the feeling of incomplete evacuation, but no gross hematuria, dysuria, abdominal distension, diarrhea, constipation, melena, or other intestinal symptoms. Prior to admission, the child was treated with antibiotics at the local clinic, but the effect was not satisfactory.

**Figure 1:**
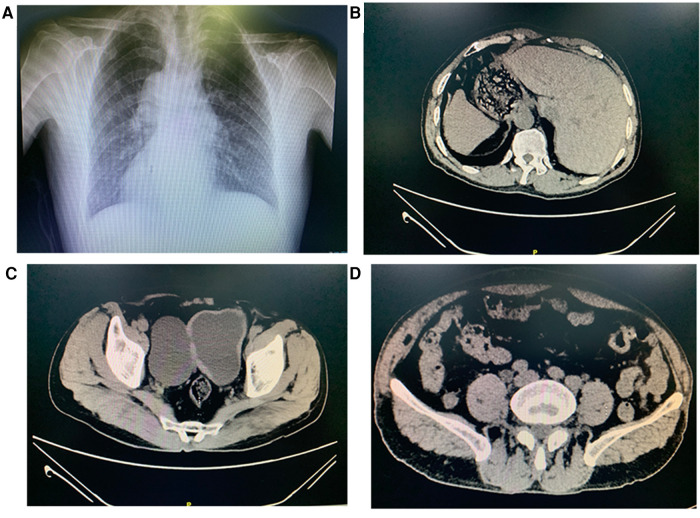
Chest radiographs show dextrocardia (**A**). Abnormal placement of stomach and liver (**B**). Computed tomography demonstrating a 78 × 50 mm diverticulum communicating with the bladder (**C**). Computed tomography demonstrating dilated hydronephrosis in the upper segment of the right-side ureteral (**D**).

The child had no specific medical history or physical deformities. There was mild pressing tenderness in the lower abdomen, which was more pronounced on the right side, but no obvious abdominal mass was observed. The urine routine was normal. Chest radiographs revealed dextrocardia (Figure 1A), and computed tomography and ultrasonography confirmed the diagnosis of SIT (Figure 1B). Pelvic computed tomography showed cystic low-density lesions on the right side of the bladder that communicated with the bladder cavity (Figure 1C), approximately 78 mm × 50 mm in size, accompanied by compression of the right pelvic segment of the ureter and dilatation of the upper segment (Figure 1D), showing the characteristics of bladder diverticulum.

Considering that the patient was quite young and the imaging did not show obvious bladder outlet obstruction, the diverticulum may have been caused by neurogenic bladder and vesicoureteral reflux. We advised the parents of the child to complete a voiding cystourethrography and urodynamic examination to confirm the diagnosis, but the parents rejected this suggestion because of economic factors.

After multidisciplinary discussion and communication with the patient's parents, laparoscopic bladder diverticulectomy was performed to relieve the ureteral compression and abdominal pain. Considering that the diverticulum was large and accompanied by SIT, to obtain sufficient operation space, the surgical team chose the transabdominal approach.

After successful anesthesia, the operation team first took the lithotomy position with indwelled urinary catheterization and filled the bladder with 200 mL of water. The orifice of the diverticulum was adjacent to the vesicoureteric junction under the ureteroscope. Therefore, we decided to insert a ureteral stent with a guide wire. Next, the patient was moved to the supine position, and the pneumoperitoneum was established. Under laparoscopic monitoring, we established a total of three channels in a fan-shaped distribution with the pubic symphysis as the center and placed the operating instruments.

**Figure 2:**
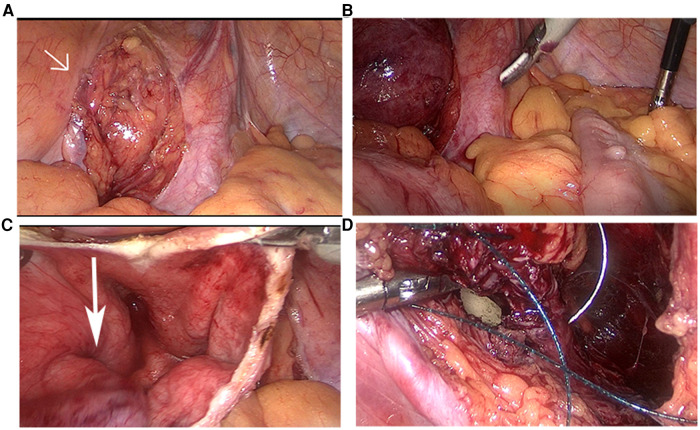
The protuberant diverticulum (arrow head) located in the right side of the bladder (**A**). The sigmoid colon located on the right side of the abdominal cavity (**B**). The narrow diverticulum orifice (arrow head) (**C**). Suture the bladder under the guidance of balloon (**D**).

After visualizing the protuberant bladder diverticulum, the peritoneum was cut open with a median incision to expose the diverticulum fully (Figure 2A). Due to the mirror location of the organs, the sigmoid colon on the right side of the bladder caused the operating space to be squeezed (Figure 2B). To avoid unnecessary injury, we added an operating channel so that the assistant could pull the sigmoid colon to the healthy side.

Next, we dissociated the diverticulum completely along the surface, cut the bladder at the upper edge of the diverticulum, absorbed the urine, and resected the diverticulum circumferentially along the diverticulum orifice (Figure 2C). Special attention was given to avoid damage to the ureteral orifice under the guidance of the ureteral stents. Under the guidance of the balloon, the bladder incision was sutured and closed continuously with a 2-0 inverted thorn suture (Figure 2D).

After no obvious urine leakage was observed by catheter injection, the peritoneum was sutured, and a pelvic drainage tube was placed. The catheter was removed 12 days after the operation. There were no obvious complications, and the patient was discharged smoothly. The diagnosis of bladder diverticulum was confirmed by pathology. The ureteral stent was removed 2 months later. The postoperative follow-up for 3 months showed that the liver and kidney function were normal, the urination symptoms were significantly improved, and there was no urinary extravasation. However, the ureter was still slightly dilated.

## Potential connections between situs inversus totalis and bladder diverticulum

Although the human body is mirror-symmetrical in outward appearance, most organs show an obvious left–right asymmetry, which is called the situs solitus (1). This asymmetry along the left–right axes originates from the left–right organizer (4), and the so-called leftward-fluid-flow is the nodal flow driven by single ciliated cells containing movable cilia. Plane cell polarization leads to backward tilting and clockwise rotation of the cilia, resulting in the inclined distribution of nodal factors to the left, which leads to a cascade of asymmetric gene expression. The signal is transmitted to the lateral plate mesoderm and then transferred to the organ primordia (5). The nodal signal transduction pathway plays a very important role, and it is considered to be the common pathway for the left–right development of embryos (1).

Changes in the genetic information that controls the assembly and function of human cilia and the Nodal signal transduction pathway can lead to lateral defects, including heterotaxy and SIT (5). SIT is generally considered to be an autosomal invisible genetic disease, and some scholars believe that the occurrence of SIT is related to defects in the function of the X chromosome (1, 6). Chromosomal abnormalities and a large number of gene mutations are thought to cause the disease. SIT is often expressed as part of syndromes such as primary ciliary dyskinesia, nephronophthisis (NPHP), polycystic kidney disease 2 (PKD2), and Bardet–Biedl (7–9). Some cases of SIT that seem to have nothing to do with ciliary dysfunction can be called isolated SIT. The environment of embryonic development and the randomness of development should be considered, such as incorrect cardiac catheter torsion (10) and some maternal factors (maternal hyperglycemia) (11).

In addition to some of the acquired diverticula secondary to elevated bladder pressure, some bladder diverticula are associated with congenital misarrangement and congenital weakness of the bladder muscle fibers; the pathological specimens often contain dysplastic muscle fibers, and the probability of malignant transformation is very low (12). Ninety percent of primary congenital bladder diverticula are located on the upper lateral side of the ureteral orifice without involving the bladder triangle, known as the Hutch diverticulum, and they are often accompanied by neurogenic bladder and vesico-ureteral reflux; adult occurrence is relatively rare (13). Bladder diverticulum can be seen in some congenital syndromes caused by genetic factors, such as *FBLN-5-*related cutis laxa (14), Williams–Bevren syndrome (15), Menkes disease syndrome, and Occipital Horn syndrome associated with *ATP7A g*ene defects (16). In addition, in some congenital diseases, patients often show multisystem involvement, and these patients are at simultaneous high risk for SIT, neurogenic bladder, and vesicoureteral reflux, which is an important consideration in terms of the pathogenesis of bladder diverticulum, especially for mirror people.

### Motile cilia diseases

Some gene mutations that control the assembly and function of motor cilia (mainly affecting the dynamic proteins of cilia) have an important effect on hydrocephalus and SIT. Dysfunction of ependymal motor cilia destroys the normal circulation of the cerebrospinal fluid, resulting in hydrocephalus. Some studies have found that cerebrospinal fluid may be related to the early occurrence of left–right asymmetry (17, 18). Mutations in *DNAI1*, *DNAI2*, *DNAH5*, and *DNAH11* may be common pathogenic factors underlying SIT and hydrocephalus (1, 19). In 1986, Jabouran reported a case of Kartagener syndrome, which is characterized by SIT and hydrocephalus (20). In 2020, Maria reported a female patient who was diagnosed with SIT and normal pressure hydrocephalus. Genetic tests revealed a new homozygous deletion in *DNAI2* (21). Normal pressure hydrocephalus is considered to be an important pathogenic factor of neurogenic bladder, which may be related to detrusor overactivity. When combined with sphincter dysfunction, bladder pressure can increase and induce bladder diverticulum. There is a lack of follow-up records for these patients, and given the above reasons, these patients may be more likely to have bladder diverticula than the general population.

### Nonmotile ciliopathies

Compared with motor cilia, nonmotor cilia lack dynein arms and usually play the role of receptors (22). However, they are also involved in many processes that affect organ laterality, such as planar cell polarization and some important signaling pathways (notch and hedgehog pathways) (23, 24). Some nonmotile ciliopathies, such as Bardet–Biedl, NPHP, and PKD syndrome, often involve multiple systems, resulting in congenital organ laterality defects (SIT and heterotaxy) and kidney disease, neurogenic bladder and vesicoureteral reflux in the urinary system (23), which are the causes of the hutch diverticulum. NPHP, Bardet–Biedl, and PKD gene mutations show clinical heterogeneity. Gene mutations at different sites produce different clinical manifestations, which depend on more comprehensive genome testing (exome sequencing) and gene targeting tests to establish genotype–phenotypic relationships.

### Caudal regression syndrome

Caudal regression syndrome (CRS) is characterized by spinal and spinal cord caudal dysplasia, malformations of the anorectal and genitourinary system, and can show congenital abnormalities such as SIT, neurogenic bladder, and vesico-ureteral reflux (25). Currarino syndrome is a special form of CRS and it is thought to be associated with mutations in the *HLXB9* gene (26), which is also considered to be key to the development and function of the normal pancreas (27). According to a recent study, 22% of CRS is associated with maternal diabetes. Maternal hyperglycemia is considered to be an important environmental factor in SIT. Yogesh reported a 4-year-old patient with SIT and vesicoureteral reflux, and his mother was diabetic (11).

### Spondylocostal dysostosis

Spondylocostal dysostosis (SCD) is a rare syndrome characterized by axial dysplasia, which can be divided into five types caused by different gene mutations in the Notch signaling pathway. SCD4 caused by *HES7* mutations can produce both SIT and meningocele (28), which can lead to detrusor overactivity and detrusor–urethral sphincter dysfunction. It can induce the occurrence of bladder diverticulum, but the incidence is not clear.

## Discussion

As mentioned above, some patients with syndromes caused by genetic variations often have clinical heterogeneity and overlaps of clinical features. Neurogenic bladder and vesicoureteral reflux are more likely to cause bladder diverticulum in patients with nonobvious bladder outlet obstruction, especially when they are complicated with SIT. A bladder diverticulum on the right needs to be distinguished from a sigmoid diverticulum located on the right due to mirror reversal, especially if the patient has bowel complications. Giant bladder diverticulum oppressing the intestine and inverted bladder diverticulum can cause ileus and abdominal pain (29). The sigmoid diverticulum is the most common cause of colovesical fistula which is a pathological communication between the bladder and the colon that can lead to unremitting intestinal and urinary symptoms, such as fecaluria, pneumaturia, urinary tract infection, dysuria, and frequent urination (30, 31). Voiding cystourethrography and imaging urodynamic examination are the “gold standard” for diagnosis (32). The internal arrangement of organs is usually indicated in imaging examinations, but because parts of SIT are caused by ciliary dysfunction, high-speed video microscope analysis, nasal NO concentration, and even gene detection are all important auxiliary examinations (33). Combined with the patient's detailed medical history and family history, the surgical team can thus have a more accurate understanding of the etiology of the patient.

Active treatment of the primary disease is critical to the prognosis. Surgical treatment is required when bladder diverticulum is associated with recurrent urinary tract infection, causes oppressive symptoms, or is suspected of malignant transformation (3). Laparoscopic bladder diverticulectomy has replaced traditional open surgery as the main surgical method at present. Some studies show that laparoscopic surgery for bladder diverticulum has success rates at least equivalent to those of traditional open surgery in the pediatric population (34). In addition, laparoscopic surgery has some typical advantages, such as a shorter length of hospital stay, a smaller incision and better cosmesis, lower incidence of incisional hernia and infection, less blood loss, and less analgesic use, and because of the high definition magnification effect of the endoscope, it has an irreplaceable role in intraoperative exploration, which is conducive to distinguishing diverticulum from surrounding tissues and reducing surgical complications (35–37).

For this child, due to the preoperative consideration of benign lesions and large diverticulum, to obtain a larger surgical space, the surgical team chose the transabdominal approach. For patients with SIT, the following points should be emphasized: first, due to the mirror distribution of the organs and frequent organ variations, precise preoperative planning should be made for the surgical team, such as the problem of the position, the selection of puncture holes, and the placement of the instruments. Although the bladder is located on the central axis, some abnormal anatomical structures around the diverticulum should be considered during the process of exposure. In this case, because the patient's sigmoid colon was located on the right side and the diverticulum was large, the assistant had to carefully pull the sigmoid colon when separating and suturing the right diverticulum so that the surgeon could obtain sufficient operating space to avoid iatrogenic fistulas between the bladder and sigmoid colon.

Second, patients with SIT are often complicated with renal dysplasia, ureteral duplication, polycystic kidney, decreased renal function, and are easily complicated with a hutch diverticulum (the ureteral orifice is close to the diverticulum), so special emphasis should be placed on the protection of the ureteral orifice and the identification of the diverticulum, such as inserting a 6Fr JJ stent at the beginning of the operation, placing the catheter parallel guide wire into the diverticulum and dilating the balloon, injecting methylene blue into the diverticulum, or injecting a fluorescent tracer into the bottom of the diverticulum (38). Ureteral bladder reimplantation should be considered when there is severe vesicoureteral reflux or when the ureteral orifice is located in the diverticulum (39).

Finally, 10% of patients with SIT have abnormal development of the gastrointestinal tract (intestinal malrotation, abnormal mesenteric blood supply, persistent descending mesocolon) (40); therefore, when the surgeon chooses the transabdominal approach, the peritoneum should be closed at the end of the operation to prevent intestinal obstruction, volvulus, and colovesical fistula but also to avoid urine entering the abdominal cavity in cases of postoperative urine extravasation.

## Conclusion

SIT with bladder diverticulum is clinically rare, and many of these cases are caused by genetic factors. There is a potential causative connection between the two conditions, and they may coexist in some congenital syndromes. Although we have found no clear evidence of the underlying genetic variation, in this case, surgeons should pay attention to the etiological diagnosis (especially in children), perform a thorough examination, and make targeted plans before surgery to meet the challenges of this special combination. In general, laparoscopic bladder diverticulectomy is safe for patients with SIT.

## Data Availability

The original contributions presented in the study are included in the article/Supplementary Material, further inquiries can be directed to the corresponding author/s.
